# Recent advances in the electrochemical construction of heterocycles

**DOI:** 10.3762/bjoc.10.303

**Published:** 2014-12-03

**Authors:** Robert Francke

**Affiliations:** 1 Department of Chemistry, University of Rostock, Albert-Einstein-Str. 3a, 18059 Rostock, Germany

**Keywords:** anodic cyclization, electrosynthesis, heterocycle, olefin coupling, organic electrochemistry, radical cyclization

## Abstract

Due to the fact that the major portion of pharmaceuticals and agrochemicals contains heterocyclic units and since the overall number of commercially used heterocyclic compounds is steadily growing, heterocyclic chemistry remains in the focus of the synthetic community. Enormous efforts have been made in the last decades in order to render the production of such compounds more selective and efficient. However, most of the conventional methods for the construction of heterocyclic cores still involve the use of strong acids or bases, the operation at elevated temperatures and/or the use of expensive catalysts and reagents. In this regard, electrosynthesis can provide a milder and more environmentally benign alternative. In fact, numerous examples for the electrochemical construction of heterocycles have been reported in recent years. These cases demonstrate that ring formation can be achieved efficiently under ambient conditions without the use of additional reagents. In order to account for the recent developments in this field, a selection of representative reactions is presented and discussed in this review.

## Introduction

The construction of heterocyclic cores undoubtedly represents a highly important discipline of organic synthesis. The large interest in this field is attributable to the occurrence of heterocyclic units in numerous natural products and biologically active compounds such as hormones, antibiotics and vitamins [1]. Considering the fact that more than 70% of all active ingredients in pharmaceutical and agrochemical products contain at least one heterocyclic unit, the particular importance of heterocyclic compounds becomes clear [2].

Most of the classical methods for heterocycle synthesis are based on the use of acids or bases at elevated temperatures, conditions which are often not compatible with the presence of certain functional groups [3–4]. In the last decades, research in this field has therefore been focused on the development of more efficient and selective strategies. In the current focus of heterocycle synthesis are C,H-activation with transition metal catalysts [5–8], oxidative cyclization using hypervalent iodine reagents [9–12], and homogeneously or heterogeneously catalyzed multicomponent reactions [13–14]. Moreover, radical cyclizations predominantly conducted using Bu_3_SnH in the presence of azobisisobutyronitrile (AIBN) play a crucial role [15–16]. However, all these methods require the use of an expensive catalyst and/or toxic and hazardous reagents. In order to meet increasing environmental and economic constraints, further efforts should be directed towards the development of mild and reagent-free methods [17–18]. In this context, electroorganic synthesis can provide an interesting and practical alternative to conventional methods for heterocycle synthesis [19–20]. Since toxic and hazardous redox reagents are either replaced by electric current (direct electrolysis) or generated in situ from stable and non-hazardous precursors (indirect electrolysis), electrosynthesis is considered to be a safe and environmentally friendly methodology [21–25]. A further interesting feature is that electrochemical reactions are feasible under very mild conditions; since the reaction rate is determined by the electrode potential, reactions with high activation energies can be conducted at low temperatures.

The electrochemical synthesis of heterocyclic compounds can be considered as a mature discipline. The last comprehensive review dealing with electrochemical heterocycle generation has been published in 1997 by Tabaković [26]. Earlier reviews on different aspects of the electrochemistry of heterocyclic compounds are also available [27–29]. However, recent innovations in electrosynthesis such as the cation pool method or the development of novel electron transfer mediators also have a significant impact on heterocyclic chemistry [30–31]. This review focuses upon both anodic and cathodic processes that lead to the formation of heterocyclic structures in view of these recent developments. Its scope is to highlight advances since the appearance of Tabacovićs review in 1997. The intention is rather to provide the reader with a general insight than to give an exhaustive overview.

## Review

**Figure 1:**
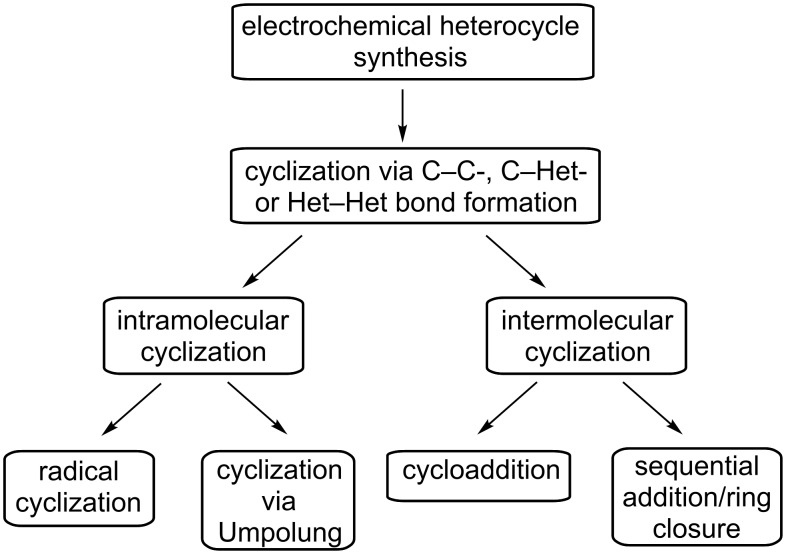
Common types of electrochemically induced cyclization reactions.

Numerous mechanistic pathways to the formation of heterocycles have been described and for a detailed treatment, interested readers are referred to earlier reviews by Lund and Tabaković [26–28]. The electrochemical heterocycle synthesis can principally proceed through C–Het-, C–C- and Het–Het-bond formation, whereby the former two represent the predominant case in recent literature (Figure 1). Furthermore, one can distinguish between intramolecular and intermolecular cyclization. The intramolecular version typically involves two functional groups linked by a tether. The electrochemical reaction leads to an Umpolung of the functional group with the lower redox potential, triggering the ring-closure reaction between a nucleophilic and an electrophilic site. Another possibility for an intramolecular ring closure is represented by electrochemically induced radical cyclization. Intermolecular cyclizations generally fall into two further categories. In the first scenario, an anodically generated nucleophile (cathodically generated electrophile) reacts with an electrophile (nucleophile) present in solution. Consequently, an intermediate is formed, which undergoes ring-closure reaction. The second scenario involves the electrochemical formation of a reactive species followed by cycloaddition in a concerted mechanism.

The examples presented hereafter are classified according to the reaction type rather than to the resulting type of heterocycle. Among the intramolecular reactions, recent efforts in electrochemical heterocycle synthesis can mostly be differentiated into anodic olefin coupling (section 1.1), radical cyclization (section 1.2), and trapping of anodically generated iminium/alkoxycarbenium ions (section 1.3). On the other hand, cycloadditions (section 2.1), sequential Michael addition/ring closure with in situ generated quinones (section 2.2) and sequential cyclizations involving acyliminium species and alkoxycarbenium ions (section 2.3) represent the majority of recently reported intermolecular electrochemical cyclizations. Cases which do not fall into any of these categories are discussed in sections 1.4 and 2.4.

**Scheme 1:**
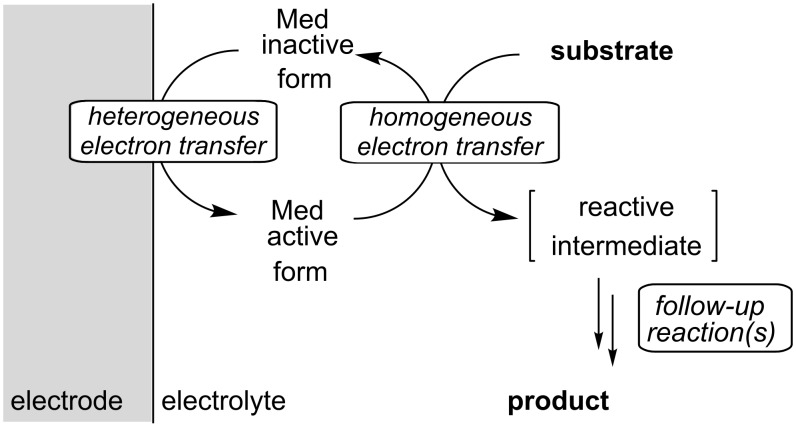
Principle of indirect electrolysis.

A further important aspect is the type of electron transfer involved in the reaction. With regard to heterocycle synthesis, both direct electrolysis involving heterogeneous electron transfer between electrode and substrate as well as indirect electrolysis using electron transfer mediators (Scheme 1) play an important role. With regard to selectivity, the direct method is often complementary to typical chemical oxidations and reductions, since electrochemical oxidation or reduction proceeds via discrete electron transfer steps rather than atom transfer. In contrast, the indirect process can either be initiated with a discrete electron transfer (outer-sphere mechanism) or proceed via bond formation (inner-sphere mechanism), depending on the type of mediator [31]. In both cases, the electrode reaction proceeds at such a low potential that the substrate is electrochemically inactive. In many cases, undesired side-reactions can be avoided by using redox mediators, since reactive intermediates do not accumulate on the electrode surface. Moreover, the indirect approach is often used in order to inhibit electrode passivation caused by formation of polymer films. In the context of heterocycle synthesis, a number of mediators based on organic molecules, inorganic salts and metal complexes have been used recently and their use will be discussed later on the basis of the relevant examples.

### 1 Intramolecular cyclizations

#### 1.1 Anodic olefin coupling

**Scheme 2:**
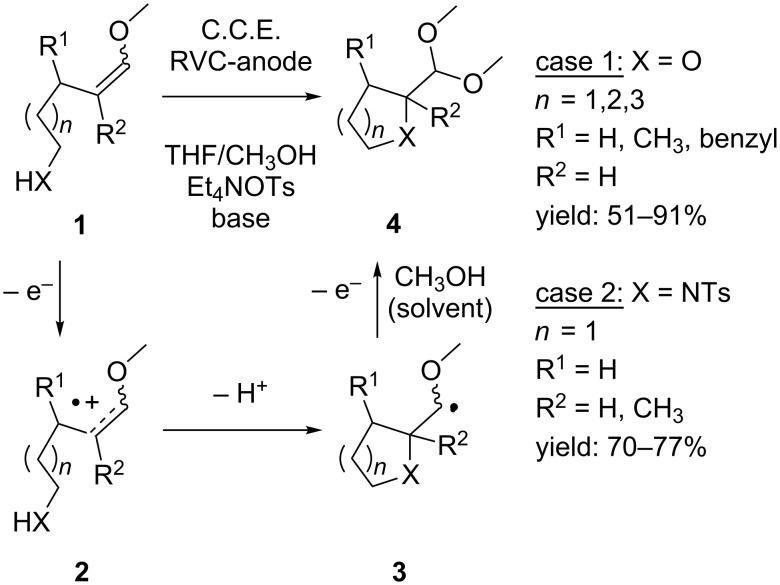
Anodic intramolecular cyclization of olefines in methanol.

The anodic oxidation of electron-rich olefins such as enol ethers **1** in methanolic solution generates radical cation **2** which can be used for a number of cyclization reactions (Scheme 2) [32–33]. Moeller et al. demonstrated that by intramolecular trapping of this highly reactive intermediate with a tethered alcohol nucleophile, a variety of tetrahydrofuran, tetrahydropyran and oxepane structures **4** can be synthesized (Scheme 2, X = O) [34]. The reaction is initiated by single electron oxidation to generate intermediate **2**, which after cyclization and deprotonation gives radical **3**. Further oxidation results in the formation of a cationic species which is trapped by methanol to yield product **4**.

This cyclization method is not restricted to hydroxy groups as trapping agents. More recently, Moeller and Xu reported that *N*-nucleophiles such as the tosylamine group can efficiently trap **2**-type radical cations, resulting in *N*-tosylated pyrrolidine products (Scheme 2, X = NHTs) [35–36].

The reactions can be carried out under galvanostatic conditions (C.C.E. = constant current electrolysis) at room temperature in an undivided cell using a vitreous carbon anode. The presence of a proton scavenger is necessary in order to obtain reasonable reaction rates. When the radical cation is trapped with a hydroxy group, the use of 2,6-lutidine is sufficient. However, a stronger base such as NaOMe is needed when tosylamines are converted in order to facilitate the cyclization reaction and to suppress intermolecular coupling. In addition to enol ethers **1**, vinyl sulfides and ketene acetals have successfully been cyclized according to Scheme 2 [34–36].

**Scheme 3:**
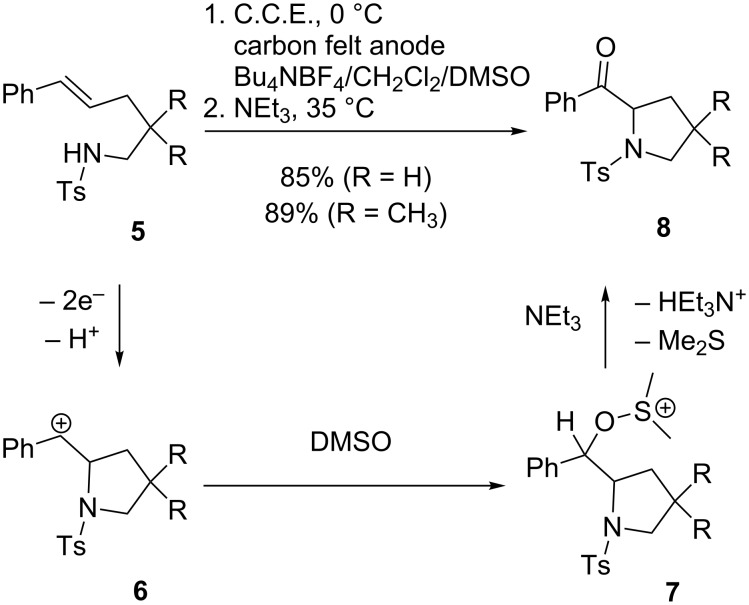
Anodic cyclization of olefines in CH_2_Cl_2_/DMSO.

An interesting modification of this anodic coupling method was achieved by Yoshida, Nokami and co-workers using the “cation pool” concept [37–39]. In this approach, the anodic oxidation of olefins was combined with a sequential chemical oxidation in a one-pot fashion (Scheme 3) [39]. By using DMSO instead of methanol as nucleophilic co-solvent for electrolysis, a pool of alkoxysulfonium ions **7** is generated from tosylamine **5**. The generation of the cation pool has to be carried out at 0 °C in order to stabilize the reactive alkoxysulfonium species. Analogously to Swern- and Moffat-type reactions, this key intermediate is then converted to ketone **8** by quenching with NEt_3_ at slightly elevated temperatures under elimination of dimethyl sulfide.

Alternatively, a tethered carboxy group can be used as the nucleophilic component, leading to the formation of lactone rings [39]. A further option is the hydrolysis of alkoxysulfonium species **7** with aqueous NaOH under formation of the corresponding secondary alcohol [40].

**Scheme 4:**
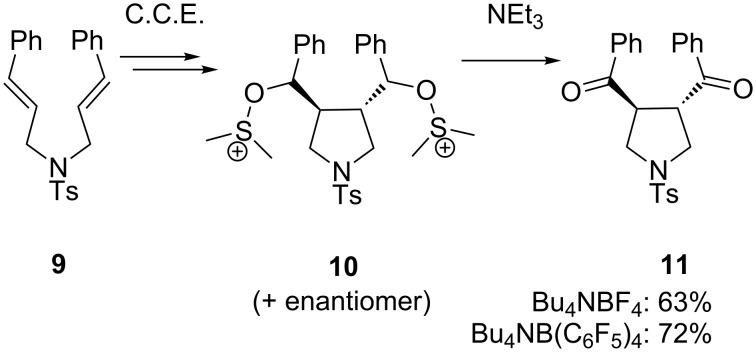
Intramolecular coupling of 1,6-dienes in CH_2_Cl_2_/DMSO.

The idea of integrating a chemical oxidation into the anodic cyclization of olefins was extended to intramolecular coupling of 1,6-dienes **9** (Scheme 4) [39]. In this version of the combined cyclization/oxidation, the second carbon–carbon double bond acts as the nucleophile after anodic oxidation, leading to bisalkoxysulfonium species **10**. When the reaction conditions depicted in Scheme 3 are applied, including quenching with NEt_3_, *exo–exo*-cyclization product **11** is obtained in 63% yield with 100% *trans*-selectivity. The yield can be increased to 72% when Bu_4_NB(C_6_F_5_)_4_ is used as supporting electrolyte.

#### 1.2 Electrochemically induced radical cyclizations

**Scheme 5:**
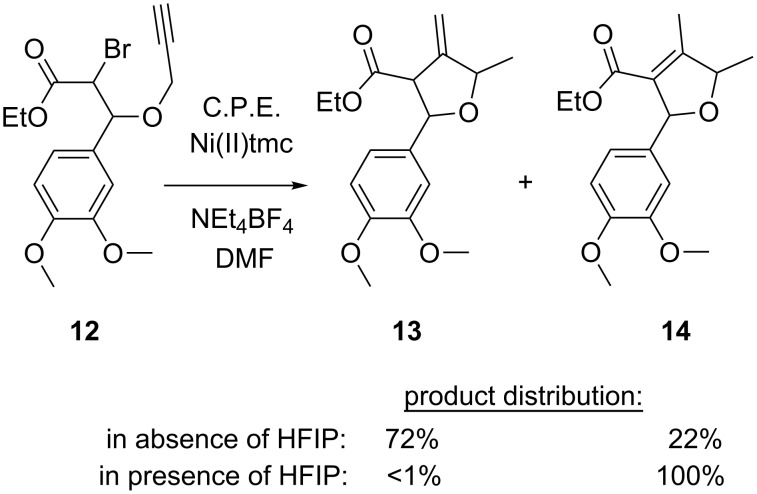
Cyclization of bromopropargyloxy ester **12**.

Among the numerous existing radical cyclization methods, the conversion of unsaturated alkyl halogenides represents one of the key-reactions for the synthesis of natural products containing aliphatic heterocycles. Such ring-closing reactions are frequently carried out using toxic tri-*n*-butyltin hydride in combination with a radical initiator such as AIBN. Peters and co-workers described an electrochemical alternative using cathodically generated nickel(I) complexes as mediators [41–42]. Under potentiostatic conditions (C.P.E. = controlled potential electrolysis) in a divided cell, bromopropargyloxy ester **12** was converted using [Ni(tmc)]Br_2_ (tmc = 1,4,8,11-tetramethylcyclam) as catalyst, leading to cyclization products **13** and **14** (Scheme 5). When **12** was electrolyzed in absence of mediator, significantly lower yields were obtained [42].

**Scheme 6:**
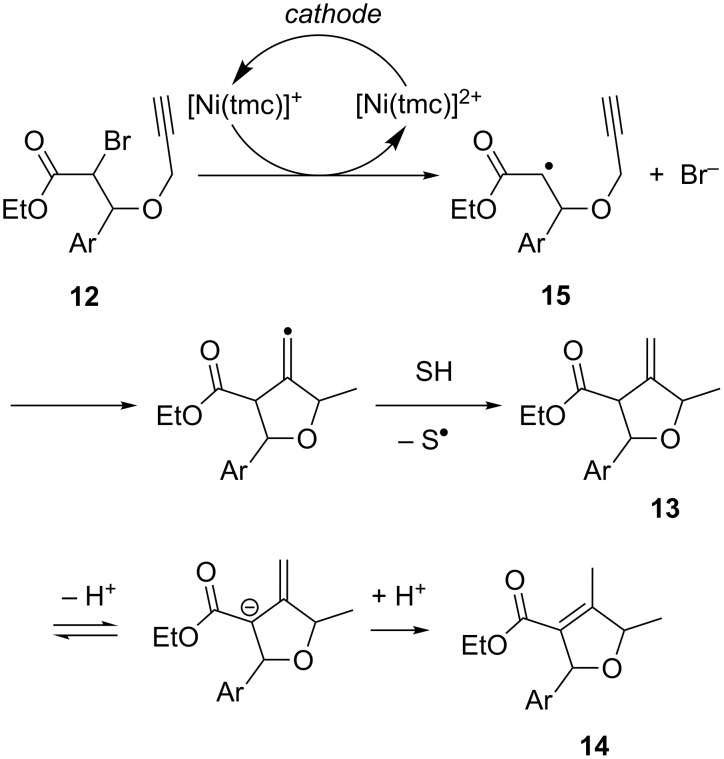
Proposed mechanism for the radical cyclization of bromopropargyloxy ester **12**.

The acidity of the reaction medium strongly influences the product distribution. Under aprotic conditions, the formation of **13** is favored, whereas in presence of HFIP (1,1,1,3,3,3-hexafluoroisopropanol) as a proton donor, product **14** is formed exclusively. On the basis of faradaic yields, cyclic voltammetry data and product distribution, the authors proposed the mechanism shown in Scheme 6. The sequence starts with electron transfer from cathodically generated [Ni(tmc)]^+^ to **12**, triggering the cleavage of the C–Br bond in the rate-determining step and resulting in radical species **15**. After rapid intramolecular cyclization, followed by hydrogen atom abstraction from the solvent, product **13** is afforded, which equilibrates in the presence of a proton donor to form the more stable α,β-unsaturated product **14**.

**Scheme 7:**
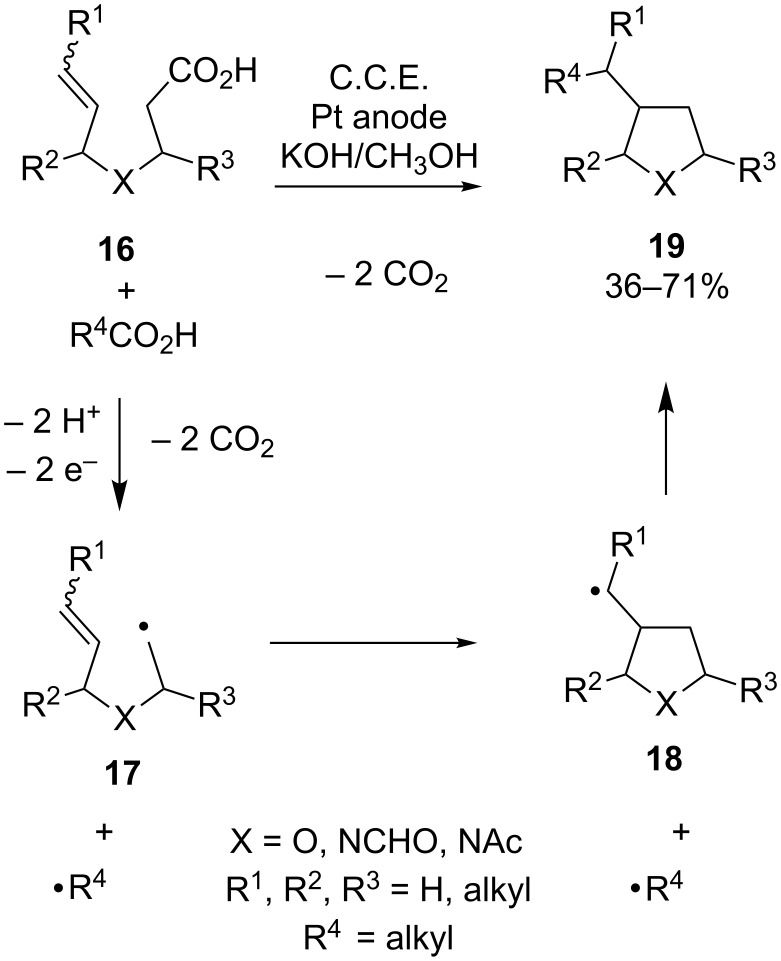
Preparation of pyrrolidines and tetrahydrofurans via Kolbe-type electrolysis of unsaturated carboxylic acids **16**.

A different radical cyclization method for the synthesis of tetrahydrofurans and pyrrolidines was developed earlier by Schäfer and co-workers [43–45]. In their approach, unsaturated and saturated carboxylic acids were simultaneously subjected to a mixed Kolbe-type oxidation in a KOH/methanol electrolyte using an undivided cell under galvanostatic conditions (Scheme 7). The cyclization reaction is initiated with the generation of radical **17** upon anodic oxidation of the potassium salt of **16**. Rapid cyclization gives **18**, which recombines with the alkyl radical R^4•^ under formation of product **19**. A synthetic challenge is represented by the competing recombination of intermediate **17** with R^4•^, resulting in an open structure which was also isolated in some cases. With unsubstituted substrate **16** (R^1^ = R^2^ = R^3^ = H) this side reaction leads to the formation of a significant amount of the undesired byproduct. However, increasing alkyl substitution leads to improved yields of the cyclization product; apparently, the cyclization rate is significantly enhanced in this case due to the Thorpe–Ingold effect [46].

**Scheme 8:**
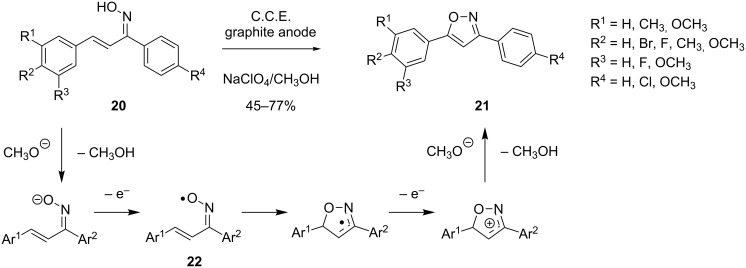
Anodic cyclization of chalcone oximes **19**.

Recently, Zeng, Little and co-workers reported a new electrochemical method for the preparation of 3,5-disubstituted isoxazoles from chalcone oximes **20** (Scheme 8) [47]. The electrolysis of **20** is carried out in an undivided cell under galvanostatic conditions using a NaClO_4_/CH_3_OH electrolyte. The cyclization is proposed to proceed via iminoxyl intermediate **22**, which is generated through deprotonation by cathodically generated methanolate and subsequent anodic oxidation. Cyclization of radical **22** is followed by further oxidation and proton abstraction to afford isoxazol **21**. The reported method features a simple setup, mild conditions (room temperature, low concentration of base) and a broad scope.

#### 1.3 Cyclization of alkoxycarbenium and iminium intermediates

**Scheme 9:**
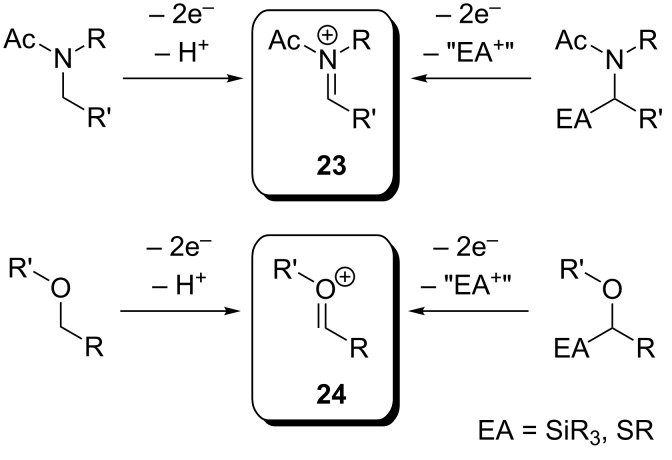
Generation of *N*-acyliminium (**23**) and alkoxycarbenium species (**24**) from amides and ethers with and without the use of electroauxiliaries.

A well-studied method for generation of aliphatic *N*- and *O*-heterocycles is the intramolecular nucleophilic trapping of anodically formed iminium (**23**) or alkoxycarbenium species (**24**). The reactive intermediates can be generated directly from ethers or carboxylic acid amides (Scheme 9) [32]. However, aliphatic ethers and amides generally exhibit high oxidation potentials, and a large number of functional groups are therefore not compatible with direct oxidation [48]. A milder and more selective approach is represented by the installation of an electroauxiliary (EA), a functional group which lowers the oxidation potential of the compound, in α-position to the oxygen/nitrogen [30,49]. In this context, the use of silyl, stannyl and thioether groups is generally preferred, since these groups are typically cleaved off upon anodic oxidation. A number of intramolecular cyclizations of **23**- and **24**-type intermediates have been reported both in presence and absence of electroauxiliaries.

**Scheme 10:**
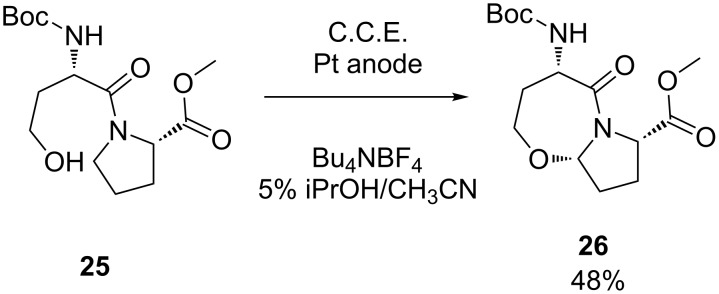
Anodic cyclization of dipeptide **25**.

In the course of their research on functionalized peptidomimetics, Moeller and co-workers found that the amide unit provides an excellent opportunity for oxidative modification of the peptide framework [50–51]. In order to construct constrained peptidomimetics, several electrochemical protocols for generation and cyclization of *N*-acyliminium species have been developed, resulting in the synthesis of a number of lactams and lactam-derived heterocycles [52]. One representative example is depicted in Scheme 10, where dipeptide **25** cyclizes via intramolecular nucleophilic attack of the hydroxy group on the anodically generated *N*-acyliminium unit [53].

**Scheme 11:**
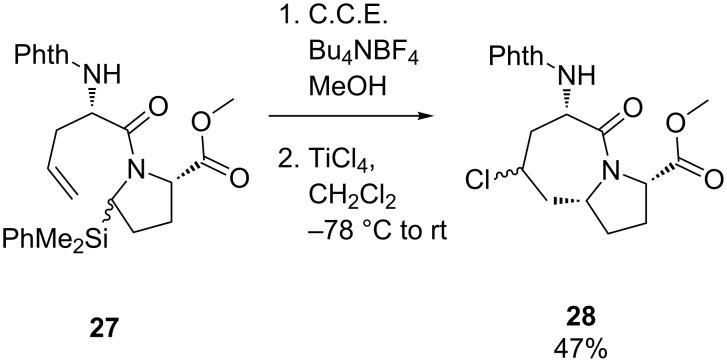
Anodic cyclization of a dipeptide using an electroauxiliary.

While this reaction proved to be very useful for the cyclization of simple amino acid derivatives, major limitations were encountered when more complicated systems were oxidized [52]. As outlined before, amide groups exhibit rather high oxidation potentials in the order of 1.95–2.10 V vs. Ag/AgCl, and their oxidation becomes therefore less selective with increasing electron-donating character of functional groups attached to the peptide. In this context, the introduction of electroauxiliaries to the peptidomimetic structures and their use for site-selective oxidation was explored [52]. The cyclization of dipeptide **27** as a model reaction is shown exemplarily in Scheme 11. Since the tethered olefin group is easier to oxidize than the amide unit, a dimethylphenylsilyl group was introduced in α-position to the amide and served as electroauxiliary. The cyclization was then accomplished in two steps, starting with the anodic oxidation of **27** in methanolic solution under galvanostatic conditions. In the second step, the resulting α-methoxy substituted intermediate was treated with TiCl_4_ in CH_2_Cl_2_ at −78 °C to give cyclized product **28**.

**Scheme 12:**
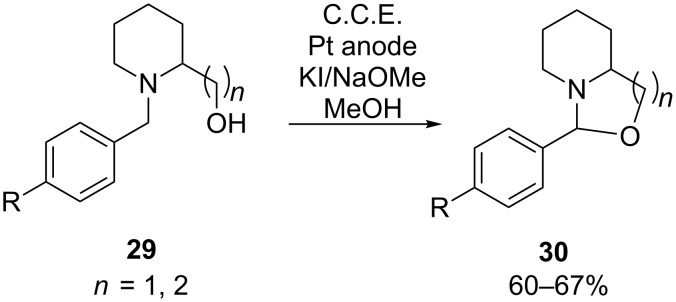
Anodic cyclization of hydroxyamino compound **29**.

Generally, the anodic oxidation of aliphatic amines leads to the formation of radical anions which can undergo multiple reaction pathways, typically leading to complex product mixtures [54]. The anodic generation of iminium species from amines for a nucleophilic α-substitution analogously to acyliminium intermediates **23** is therefore a rare case. However, Okimoto et al. could recently demonstrate that iminium species can be generated selectively and used for a cyclization reaction when a stabilizing benzyl group is attached to the nitrogen (Scheme 12, compound **29**) [55]. The hydroxy group tethered to the substrate serves for nucleophilic trapping of the iminium species under formation of oxazolidine or 1,3-oxazinane species **30**. According to their protocol, **29** is electrolyzed under galvanostatic conditions in a divided cell, using a NaOMe/MeOH electrolyte and potassium iodide as electron transfer mediator. The method provides access to a number of 2-aryl-1,3-oxazolidines and 2-aryl-1,3-oxazinanes.

**Scheme 13:**
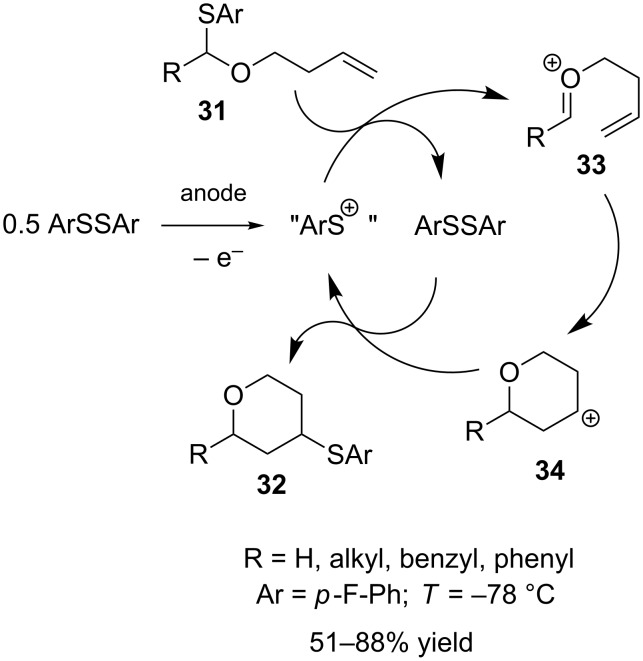
Cyclization of unsaturated thioacetals using the ArS(ArSSAr)^+^ mediator.

An intriguing example for the intramolecular cyclization of alkoxycarbenium species has been reported recently by Suga, Yoshida et al. [56]. Unsaturated thioacetals **31** were converted anodically to 2,4-substituted tetrahydropyrans **32** using a mediating system that is based on the ArS(ArSSAr)^+^ species, an equivalent of ArS^+^ (Scheme 13). ArS(ArSSAr)^+^ is formed upon anodic oxidation of ArSSAr at low temperatures and can be employed for the generation of cationic intermediates (indirect cation pool method) [57]. In the presence of a substrate with a thioaryl group (**31**), “ArS^+^” is continuously regenerated (cation chain mechanism) and therefore, both ArSSAr and electric current can be employed in catalytic amounts. After formation of alkoxycarbenium ion **33**, cyclization proceeds and the resulting carbenium ion **34** is trapped by ArSSAr to give **32**.

The ArS(ArSSAr)^+^ species can either be generated prior to addition of **31** or in presence of the substrates. The use of tetrafluoroborate salts is associated with low yields due to fluorination of intermediate **34** and therefore has to be avoided. In contrast, good results are obtained when the reaction is carried out using Bu_4_NB(C_6_F_5_)_4_ as supporting electrolyte.

#### 1.4 Further intramolecular cyclization reactions

**Scheme 14:**
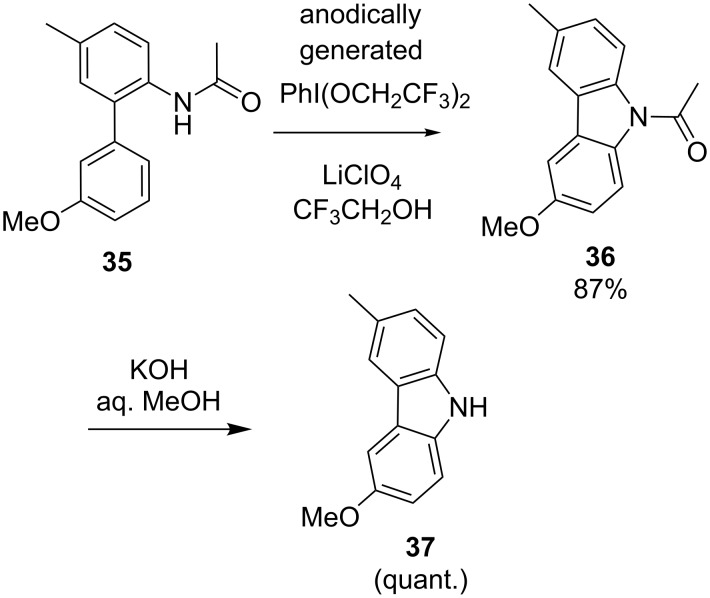
Cyclization of biaryl **35** to carbazol **36** as key-step of the synthesis of glycozoline (**37**).

**Scheme 15:**
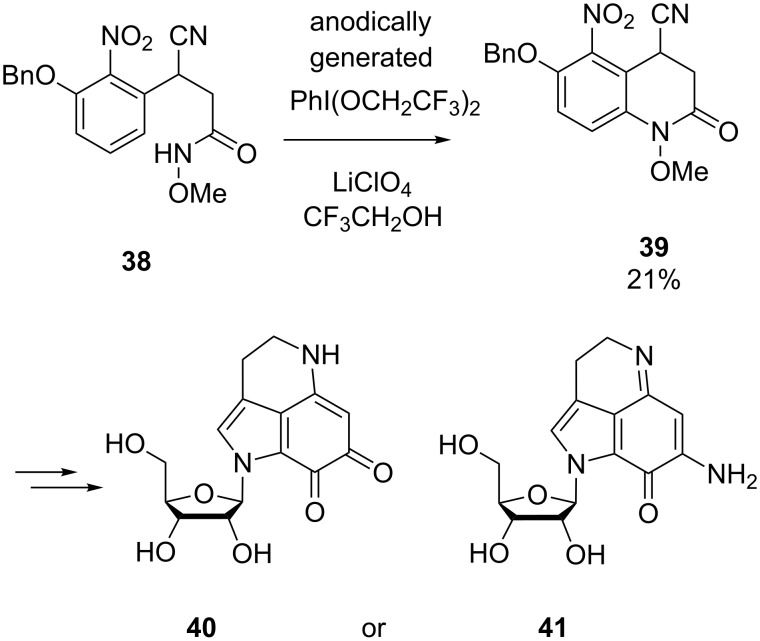
Electrosynthesis of **39** as part of the total synthesis of alkaloids **40** and **41**.

As a part of their efforts in the total synthesis of several natural products, Nishiyama and co-workers developed an electrochemical method for the construction of *N*-heterocyclic cores (see examples depicted in Scheme 14 and Scheme 15) [58–60]. PhI(OCH_2_CF_3_)_2_ was generated anodically from iodobenzene in a solution of LiClO_4_ in 2,2,2-trifluoroethanol and served as reagent for the oxidative intramolecular coupling of phenyl rings with amide or carbamate groups. With control experiments the authors demonstrated that this in situ generated reagent works more efficiently in such cyclizations than the more frequently used PIFA reagent. For instance, cyclization of biaryl **35** to carbazole **36** was achieved using this indirect electrochemical approach (Scheme 14) [59–60]. The transformation represents the key-step of the synthesis of glycozoline **37**, an antifungal and antibacterial agent. Analogously, **38** was converted to **39** as a part of the multistep synthesis of two different tetrahydropyrroloiminoquinone alkaloids **40** and **41** (Scheme 15) [58,60].

**Scheme 16:**
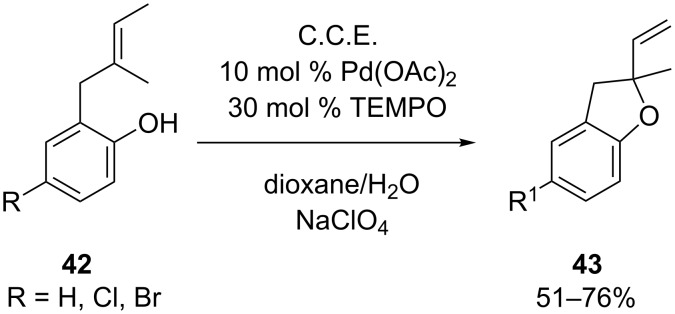
Wacker-type cyclization of alkenyl phenols **42**.

Tanaka et al. could achieve the electrochemical construction of 2,3-dihydrobenzofuran structures **43** via double-mediatory Wacker-type cyclization of alkenyl phenols **42** [61]. Pd(OAc)_2_ was used to catalyze the cyclization while TEMPO served as redox mediator for the electrochemical regeneration of the catalytically active Pd(II) species. In contrast to conventional Wacker-type cyclizations, where stoichiometric amounts of co-oxidant are employed at elevated temperatures, the electrochemical version proceeds smoothly at room temperature. In the case depicted in Scheme 16, the electrolysis was carried out in a divided cell under galvanostatic conditions using platinum electrodes. Among several electrolyte compositions, NaClO_4_ in a 7:1 mixture of dioxane/water proved to be the most efficient one. Halogen substituents on the phenol unit are tolerated under the described reaction conditions. In contrast, electron rich substrates (R = OMe) render unsatisfactory results.

**Scheme 17:**
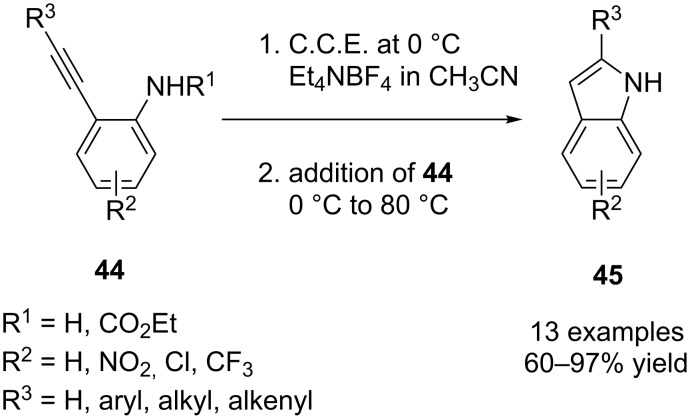
Cathodic synthesis of indol derivatives.

In view of the fact that the indole unit is present in a variety of natural products and biologically active compounds, Arcadi, Rossi et al. reported a clean and mild electrochemical method for the construction of this heterocycle (Scheme 17) [62]. The procedure is initiated with the electrolysis of the Et_4_NBF_4_/CH_3_CN electrolyte at 0 °C in a divided cell, followed by addition of **44** to the cathodic chamber after completed electrolysis (*Q* = 2.5*F*/mol). The cyanomethyl anion, a cathodically generated base which is formed upon reduction of the solvent CH_3_CN, triggers the cyclization reaction via deprotonation of the amide group. According to the authors, the deprotonation is followed by ring closure on the C–C-triple bond, generating a carbanion intermediate which is then protonated by the solvent. For sufficient cyclization rates, the reaction mixture has to be heated to 80 °C. Various alkynylanilines with different substituents on the aromatic ring and on the alkynyl group were cyclized in good to excellent yields. When *N*-ethoxycarbonyl-substituted anilines are converted under the conditions described above, the carbamate group is cleaved and unprotected indoles are obtained.

**Scheme 18:**
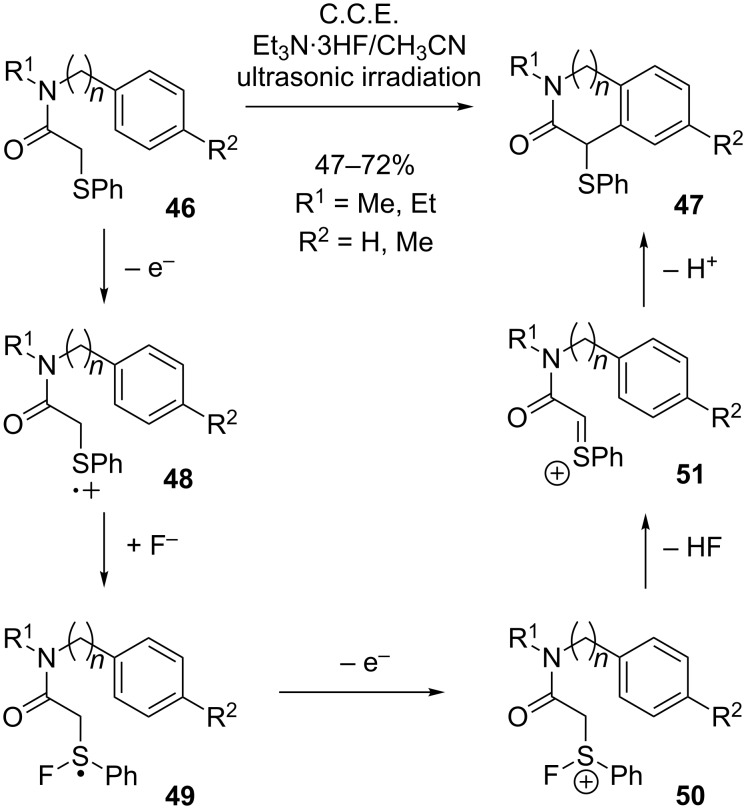
Fluoride mediated anodic cyclization of α-(phenylthio)acetamides.

An electrochemical method for the synthesis of oxindoles and 3-oxotetrahydroisoquinolines **47** via intramolecular cyclization under C–C-bond formation was reported by Atobe, Fuchigami et al. (Scheme 18) [63]. The protocol is based on the anodic oxidation of α-(phenylthio)acetamides **46** in the presence of Et_3_N∙3HF. The latter serves as supporting electrolyte and as fluoride source for mediation of the reaction. In absence of fluoride, the formation of the cyclization product was not observed. The authors proposed a mechanism, in which after initial one-electron oxidation the resulting radical cation **48** is attacked by a fluoride ion under formation of an S–F bond. Radical **49** is then further oxidized to the corresponding cationic species **50**, which undergoes elimination of HF under formation of cationic intermediate **51**. Finally, intramolecular trapping by the aromatic ring in a Friedel–Crafts type reaction leads to cyclization and formation of product **47**. A selectivity problem is caused by concurrent nucleophilic attack of fluoride ions on intermediate **51**. However, this undesired side reaction is suppressed when ultrasonic irradiation is applied during electrolysis. The authors studied the product distribution under variation of stirring speed and temperature in order to determine whether improved mass transport or strong local heating is responsible for the improved selectivity. Whereas an increase of the stirring speed did not significantly affect the ratio between desired cyclization product **47** and fluorinated byproduct, higher temperatures lead to preferential formation of the desired cyclization product. In the reported case, the use of ultrasonic radiation leads to significantly better results compared to conventional heating. These results suggest that the effect of ultrasound on the selectivity is attributable to strong local heating at the electrode surface, which increases the temperature sufficiently for cyclization. In contrast, such high temperatures are not available by conventional heating, where the reaction temperature is limited by the boiling point of the solvent.

**Scheme 19:**
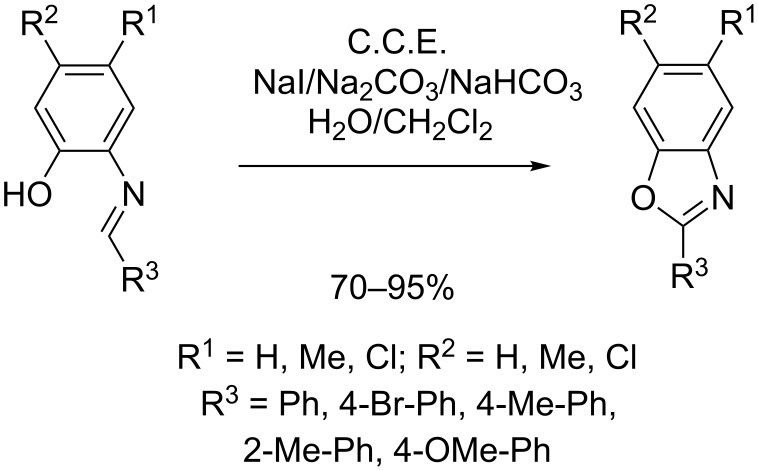
Synthesis of 2-substituted benzoxazoles from Schiff bases.

Zeng, Little et al. described an indirect electrochemical method for the generation of 2-substituted benzoxazoles from Schiff bases (Scheme 19) [64]. Using 20 mol % NaI as redox mediator, the electrolysis is conducted under galvanostatic conditions in an undivided cell. A two-phase system composed of a sodium carbonate buffer solution and dichloromethane is employed as electrolyte. The method features attractive yields (70–95%) and a broad scope with regard to substitution on the phenylene moiety (R^1^ and R^2^) and on the oxazole unit (R^3^).

### 2 Intermolecular cyclizations

#### 2.1 Cycloadditions with anodically generated intermediates

A well-established strategy for the construction of certain six-membered heterocycles is the electrochemical generation of heterodienes for Diels–Alder cycloadditions [65–66]. In this context, electrosynthesis provides a significant advantage over conventional methods: Instable diene precursors which are difficult to synthesize by conventional means, can be conveniently generated in situ at low temperatures. The electrogenerated intermediate is subjected to cycloaddition either by in situ trapping with the dienophile or by using the cation pool approach.

**Scheme 20:**
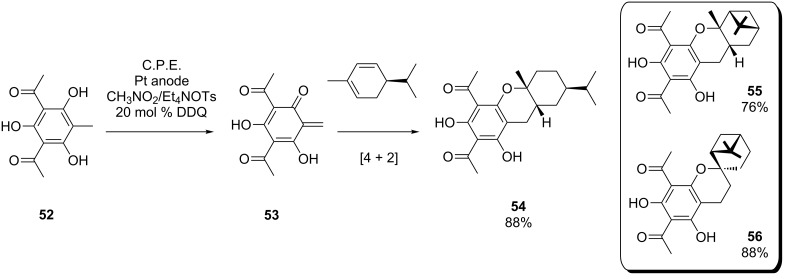
Synthesis of euglobal model compounds via electrochemically induced Diels–Alder cycloaddition.

Chiba et al. reported an interesting example for the use of such an electrochemically induced cycloaddition in natural product synthesis. The cyclization method was used for the generation of structures **54**–**56** (Scheme 20), which represent model compounds for euglobals, natural products which can be obtained by extraction of eucalyptus leaves [65]. These structures consist of a terpene element and a benzodihydropyran unit and feature antiviral activity. First, quinomethane intermediate **53** is formed upon indirect anodic oxidation of **52**, which reacts with α-phellandrene at room temperature to give compound **54**. Alternatively, **53** can be converted with α- and β-pinene to form euglobal model compounds **55** and **56**.

The electrolysis was carried out in an undivided cell under potentiostatic conditions with a CH_3_NO_2_/Et_4_NOTs electrolyte. DDQ was employed as a redox mediator, allowing for operation at a relatively low electrode potential of 0.45 V vs. SCE. Furthermore, the use of PTFE-coated platinum as working electrode proved to be beneficial. Presumably, the reaction sequence proceeds within the hydrophobic electrode coating, where the highly reactive intermediate **53** is protected from side-reactions and the reaction with the hydrophobic dienophile is facilitated.

**Scheme 21:**
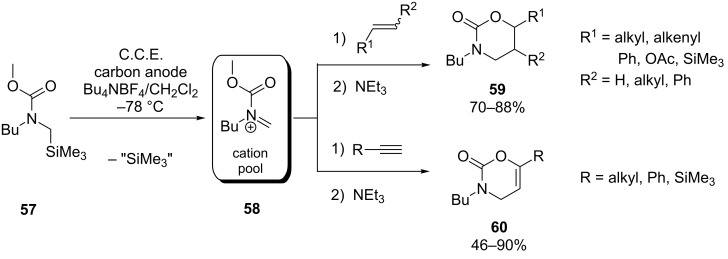
Cycloaddition of anodically generated *N*-acyliminium species **58** with olefins and alkynes.

Yoshida, Suga and co-workers reported on the use of electrogenerated *N*-acyliminium ions as heterodienes in [4 + 2] cycloadditions [66]. It was found that these highly reactive species, generated from silylated carbamate **57** at −78 °C (cation pool method), undergo cycloaddition at 0 °C with a variety of electron-rich alkenes and alkynes (Scheme 21) to afford 1,3-oxazinan-2-ones **59** and 3,4-dihydro-1,3-oxazin-2-ones **60**, respectively. The product yields strongly depend on the electronic character of the substituents on the dienophile. While monoalkyl- and monophenyl-substituted dienophiles generally render moderate to good yields, excellent results can be obtained either with silylated or acetoxylated alkenes/alkynes or with dienophiles containing two phenyl moieties. The conversion of alkyl-substituted olefins proceeds stereospecifically with respect to *E*- and *Z*-configuration, yielding either the *cis-* or *trans*-cycloadduct exclusively. Consistent with the results of computational studies conducted by the authors, this stereospecifity points towards a concerted reaction mechanism. In contrast, cycloaddition of phenyl-substituted olefins seems to proceed via a stepwise mechanism involving a cationic intermediate, since partial loss of the stereospecifity was observed.

Furthermore, the method for mixing of the cation pool with a solution of dienophile plays an important role. Best results were obtained by using a micromixer at 0 °C. While simultaneous pouring of the solutions into a flask at −78 °C still renders attractive results, addition of dienophile to **58** and vice versa leads to significantly lower yields.

**Scheme 22:**
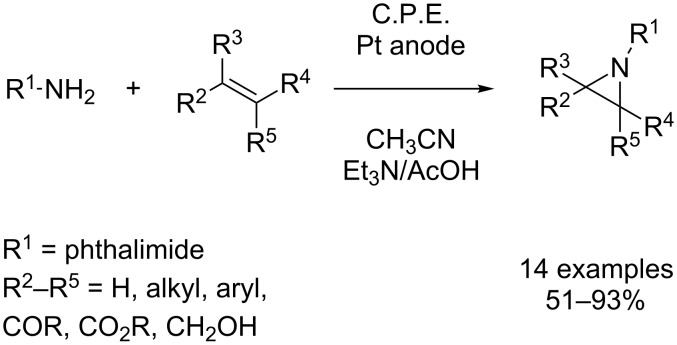
Electrochemical aziridination of olefins.

**Scheme 23:**
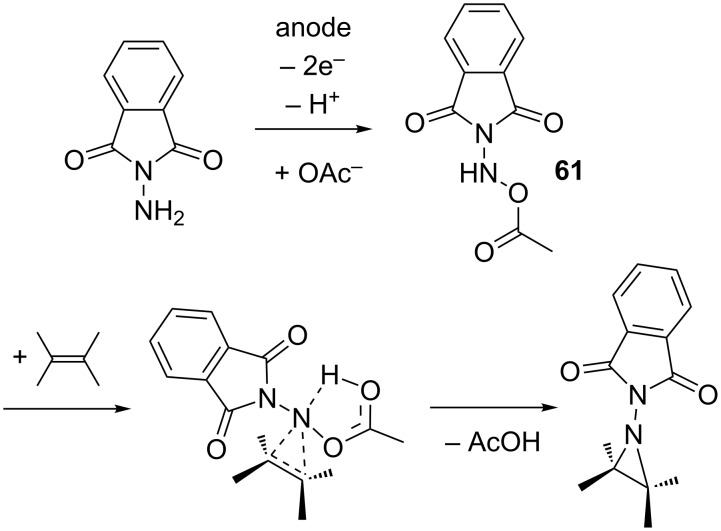
Proposed mechanism for the aziridination reaction.

Due to the synthetic usefulness of aziridines, the aziridination of olefins is of particular interest for organic chemists [3]. The three-membered aziridine ring exhibits an enormous strain and is therefore susceptible to ring-opening reactions with a variety of nucleophiles [3–4]. Such transformations lead to 1,2-heteroatom structures which are often found in pharmaceuticals and natural products. Yudin et al. described an electrochemical aziridination process where an anodically generated nitrene equivalent was transferred to a broad range of olefins using readily available *N*-aminophthalimide (Scheme 22) [67–68]. In contrast to conventional olefin aziridation, which is typically accomplished via metal-catalyzed nitrene transfer to the C–C-double bond, the electrochemical approach proceeds reagent- and catalyst-free. Both electron-rich and electron-deficient olefins can be efficiently converted. The electrolysis is carried out under potentiostatic conditions in a divided cell at room temperature using a platinum working electrode. A mixture of triethylamine and glacial acetic acid (1:1 molar ratio) in acetonitrile serves as electrolyte. The presence of acetate turned out to be crucial for aziridine formation, since with the use of other supporting electrolytes such as Bu_4_NBF_4_ the formation of the desired product could not be observed. Apparently, acetate ions are stabilizing the anodically generated nitrene species via formation of adduct **61** (Scheme 23), which then undergoes concerted addition to the olefin. The fact that upon anodic oxidation in absence of olefins, *N*-aminophthalimide dimerizes readily to the corresponding tetrazene compounds, supports the postulated intermediate **61**. Furthermore, *N*-acetoxyamino species **61** can be observed by NMR at temperatures below 5 °C. Finally, the stereospecifity of the reaction with respect to *Z*- and *E*-configuration of the olefins is a further indication for a concerted mechanism involving intermediate **61**.

#### 2.2 Anellation of in situ generated 1,2-benzoquinones via sequential Michael addition/ring closure

**Scheme 24:**
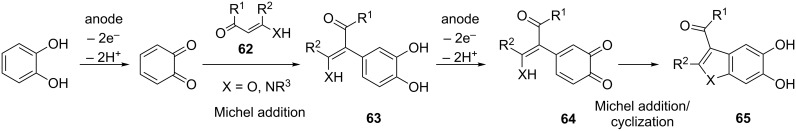
Electrochemical synthesis of benzofuran and indole derivatives.

An electrochemical method for the synthesis of benzofuran and indol derivatives is based on the oxidation of catechol in presence of 1,3-dicarbonyl compounds or analogous C,H-acidic compounds **62** (Scheme 24) [69–72]. The anodically generated 1,2-benzoquinone undergoes a Michael reaction with **62** under formation of adduct **63**, which is further oxidized to give benzoquinone **64**. Finally, anellation proceeds in a second Michael addition step under formation of heterocyclic compound **65**. In the reported cases, both the generation of 1,2-benzoquinone and the anellation reaction proceed smoothly at room temperature. In addition to the fact that the reaction is carried out without the use of oxidation agents, a further advantage of this method is the possibility for operation in an aqueous electrolyte. Typically, aqueous sodium acetate or phosphate buffer solutions are used for this type of reaction [69–72]. In many cases, the product can be obtained in high purity by simple filtration of the precipitate after completed reaction and thorough washing with water.

**Scheme 25:**
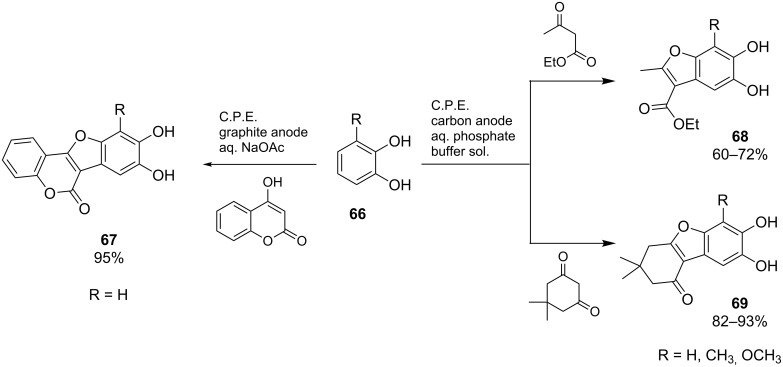
Anodic anellation of catechol derivatives **66** with different 1,3-dicarbonyl compounds.

The synthesis of 11,12-dihydroxycoumestan **67** by anodic oxidation of catechol in the presence of 4-hydroxycoumarin was reported by Tabaković et al. (Scheme 25, left) [69]. Using an undivided cell, an aqueous sodium acetate electrolyte and a graphite anode, the product was obtained in 95% yield. In a similar fashion, benzofurans of type **68** and **69** were synthesized by Nematollahi and co-workers from 3-substituted catechols **66** (Scheme 25, right) [70–71]. Interestingly, the formation of **68** and **69** proceeds under very high regioselectivity.

**Scheme 26:**
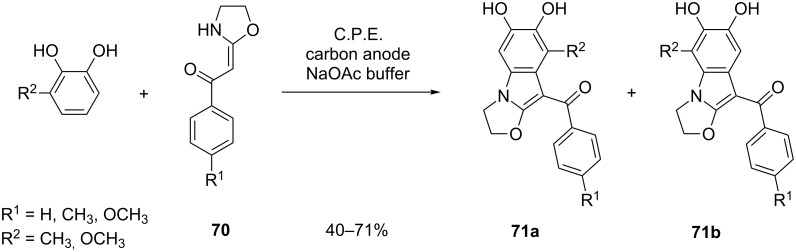
Electrosynthesis of 1,2-fused indoles from catechol and ketene *N*,*O*-acetals.

Zeng et al. developed a method for the synthesis of 1,2-fused indoles **71** (Scheme 26) based on the reaction depicted in Scheme 24 [72]. In this example, 1,2-benzoquinone derivatives are generated in the presence of ketene *N*,*O*-acetals **70**. Out of four possible regioisomers, **71a** and **71b** are exclusively formed (in ratios **71a**/**71b** between 1:1 and 1:2). In a follow-up study, unreacted α-arylated ketene *N*,*O*-acetal intermediates (**63**, Scheme 24) have been identified as byproducts [73–74]. Furthermore, it was demonstrated that ketene *N*,*S*-acetals can also be employed for indole synthesis, although the use of these substrates is associated with lower yields compared to *N*,*O*-acetals [75].

#### 2.3 Acyl iminium ions and alkoxycarbenium ions in intermolecular cyclizations

**Scheme 27:**
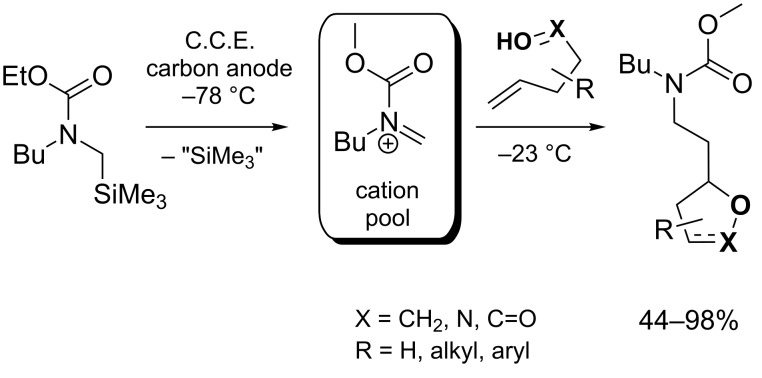
Reaction of *N*-acyliminium pools with olefins having a nucleophilic substituent.

Yoshida, Suga and co-workers investigated the electrochemical synthesis of five-membered heterocycles by integration of an intermolecular and an intramolecular step in one sequence. For this purpose, acyl iminium ions, electrogenerated as a cation pool at −78 °C, were converted at −23 °C with olefins bearing a nucleophilic group (Scheme 27) [76]. The cyclization is initiated by nucleophilic attack of the alkene on the acyl iminium species under C,C-bond formation. The resulting cationic intermediate is then trapped by the tethered nucleophile in an exo cyclization step under formation of a C,O-bond and generation of the five-membered heterocycle. Hydroxy, carboxy and oxime moieties were tested as nucleophiles for this reaction, leading to the corresponding tetrahydrofurans, γ-lactones and isoxazolines, respectively.

**Scheme 28:**
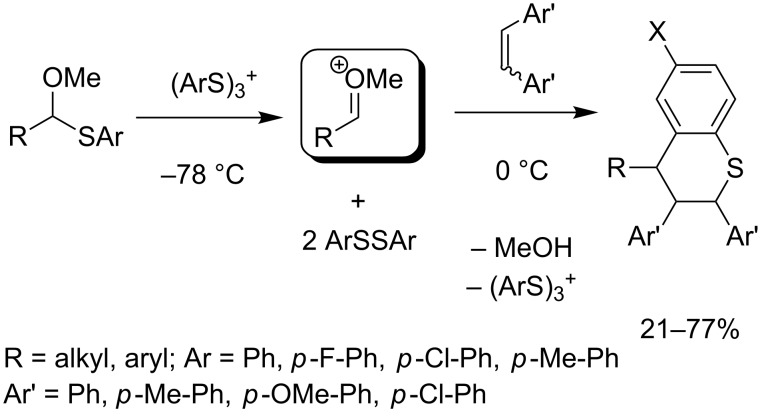
Synthesis of thiochromans using the cation-pool method.

The chemistry depicted in Scheme 13 was also used for the construction of thiochroman frameworks in a sequential intermolecular cyclization reaction (Scheme 28) [77]. A cation pool of methoxycarbenium ions is generated at −78 °C from the corresponding thioacetal using anodically formed ArS(ArSSAr)^+^, and converted at 0 °C with 4,4’-disubstituted stilbenes and ArSSAr to give the desired thiochroman. Both aliphatic (R = alkyl) and benzylic (R = Ar) thioacetals can be used, rendering the corresponding thiochroman in moderate to good yields. The reaction does not proceed stereospecifically with respect to *E*- and *Z*-configuration of the olefin. Out of four possible diastereomers, only two are obtained.

#### 2.4 Anodic oxidation of 2,4-dimethylphenol

**Scheme 29:**
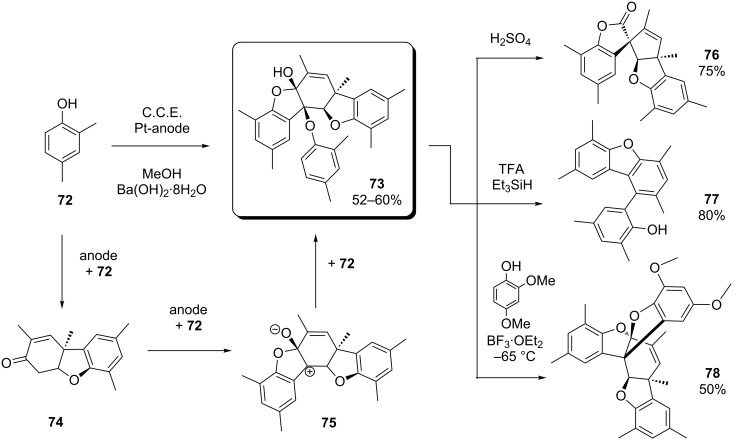
Electrochemical synthesis and diversity-oriented modification of **73**.

An interesting electrochemical route to complex scaffolds containing five membered *O*-heterocycles was found by Waldvogel and co-workers. In the course of their studies on the synthesis of 2,2’-biphenols via anodic coupling of phenols [78–79], they could observe the formation of spiropentacyclic scaffold **76** (Scheme 29) as byproduct of the electrolysis of 2,4-dimethylphenol (**72**) [80]. Later it turned out that compound **73** (a dehydrotetramer of **72**) is the actual electrolysis product, which reacted to **76** via thermal rearrangement under loss of a phenol unit during purification of the crude product by sublimation [81]. The authors proposed a mechanism where the formation of **73** starts with the anodic generation of a derivative of Pummerer’s ketone (**74**) from two phenol units. After a second oxidation step and condensation with **72**, intermediate **75** is formed. Trapping by a further phenol unit results in product **73**.

Since spiropentacycle **76** resembles the core moieties of several natural products and therefore seemed to be a useful starting point for further investigations [81], the authors decided to optimize the reaction conditions in favor of the formation of **73**. As optimum reaction conditions, the use of a Ba(OH)_2_/MeOH electrolyte in combination with platinum electrodes and a current density of 12.5 mA/cm^2^ was identified. Under these conditions, **73** precipitates in the course of the electrolysis and can be obtained in high purity by simple filtration and washing. It was also found that compared to thermal treatment, the rearrangement of **73** to **76** proceeds by far more efficiently in the presence of sulfuric acid (Scheme 29, top right).

In a follow-up study, the elaborated electrolysis protocol served as a key-step for the generation of a variety of polycyclic structures containing typical structural elements of bioactive compounds [82]. Since **73** provides manifold possibilities for structural modifications, the generation of complex and structurally diverse scaffolds could be achieved in very few steps (diversity-oriented approach) [83]. In addition to the synthesis of **76**, two more examples for such diversity-oriented transformations are depicted in Scheme 29 (middle right and bottom right): Structures **77** and **78** were obtained upon treatment with TFA/Et_3_SiH and BF_3_∙OEt_2_/2,4-dimethoxyphenol, respectively.

## Conclusion

Undoubtedly, much progress has been made in the electrochemical synthesis of heterocyclic compounds since Tabaković's review appeared in 1997. Advances in anodic olefin coupling or electrochemically induced radical cyclization have made important contributions to this field. Moreover, the emergence of the cation-pool method has significantly expanded the toolbox of the electrochemist with regard to the synthesis of heterocyclic compounds. In many cases, the unique selectivity of electrochemical transformations could successfully be utilized for the construction of heterocyclic cores within natural products. It was also demonstrated that electrosynthesis can be a useful method for the generation of complex heterocyclic scaffolds from very simple precursors in a single step.

Obviously, electrosynthesis provides a number of possibilities for the construction of heterocyclic cores. In many cases, electrochemistry represents a complementary method to conventional synthesis, due to unique selectivity and the possibility for electrochemical Umpolung. However, considering the fact that heterocycle-containing compounds represent the major part of active ingredients in pharmaceutics and crop protection, a lack of efficient protocols for the electrosynthesis of such compounds becomes evident. In particular, the electrochemical construction of aromatic heterocycles deserves more attention, since they represent the larger portion of drugs [1]. Moreover, most of the electrochemical heterocycle syntheses reported so far were achieved by anodic oxidation, whereas cathodic heterocycle generation seems to represent a rare case. Of course this means that there are unexplored territories and numerous opportunities for research in this field.

**Table 1:** List of abbreviations.

Abbreviation	Explanation

C.C.E.	constant current electrolysis
C.P.E.	constant potential electrolysis
AIBN	azobisisobutyronitrile
tmc	1,4,8,11-tetramethylcyclam
DMSO	dimethyl sulfoxide
HFIP	1,1,1,3,3,3-hexafluoroisopropanol
EA	electroauxiliary
DDQ	2,3-dichloro-5,6-dicyano-1,4-benzoquinone
SCE	saturated calomel electrode
PTFE	polytetrafluoroethylene
PIFA	[bis(trifluoroacetoxy)iodo]benzene
TFA	2,2,2-trifluoroacetic acid
TEMPO	2,2,6,6-tetramethylpiperidine-1-oxyl
